# Impact of early diagnosis of carotid artery stenosis by carotid ultrasound: A protocol of systematic review and meta-analysis

**DOI:** 10.1097/MD.0000000000019709

**Published:** 2020-05-29

**Authors:** Li-wei Qin, Li-hong Qin, Yun Yu, Xin-wei Hou, Chen Wang, Christina Weeks

**Affiliations:** a Department of Physical Diagnosis; b First Ward of Neurology Department; c Department of Education; d School of Clinical Medicine, Jiamusi University; e Second Ward of Neurology Department, First Affiliated Hospital of Jiamusi University, Jiamusi, China; f Medical School, University of Edinburgh, Edinburgh, UK.

**Keywords:** carotid artery stenosis, carotid ultrasound, early diagnosis, sensitivity, specificity

## Abstract

**Background:**

The purpose of this study is to explore the impact of carotid ultrasound (CU) for early diagnosis of carotid artery stenosis (CAS).

**Methods:**

Literatures will be sought from the following electronic databases: MEDLINE, EMBASE, Cochrane Library, PSYCINFO, Web of Science, Allied and Complementary Medicine Database, and China National Knowledge Infrastructure. The search will cover from the start of indexing to the present without any limitations of language and publication status. All study quality will be assessed by Quality Assessment of Diagnostic Accuracy Studies tool, and data will be analyzed by RevMan V.5.3 software and Stata V.12.0 software.

**Results:**

This study will investigate the impact of CU for early diagnosis of CAS through sensitivity, specificity, positive likelihood ratio, negative likelihood ratio, and diagnostic odds ratio.

**Conclusion:**

The findings of this study may provide helpful evidence for the impact of CU for early diagnosis of CAS.

**Systematic review registration:**

PROSPERO CRD42019153904.

## 1 Introduction

Carotid artery stenosis (CAS) is an important cause of ischemic stroke worldwide,^[1–3]^ which affects >600,000 American adults each year.^[4]^ It has been estimated that about 7% to 12% of all strokes and 9% to 15% of all ischemic strokes result from advanced CAS.^[5]^ A variety of risk factors can lead to this condition, such as smoking, hyperlipidemia, male sex, and age.^[6–9]^ It is very important to diagnose CAS at early stage. Previous studies have reported that carotid ultrasound (CU) can help to detect CAS.^[10–21]^ However, no study has systematically investigated the impact of CU for early diagnosis of CAS. Therefore, this study will comprehensively and systematically assess the impact of CU for early diagnosis of CAS.

## 2 Methods and design

### 2.1 Study registration

This study has been funded and registered on PROSPERO (CRD42019153904). It has been reported according to the guidelines of Preferred Reporting Items for Systematic Review and Meta-Analysis Protocols Statement.^[22]^

### 2.2 Inclusion criteria for study selection

#### 2.2.1 Type of studies

All case-controlled studies on checking the impact of CU for early diagnosis of CAS will be included in this study. All other studies, such as experimental study, non-clinical study will all be excluded.

#### 2.2.2 Type of participants

In this study, we will consider the reports of patients with angiocardiography-proven CAS for inclusion.

#### 2.2.3 Type of index test

Index test: All participants who received CU will be received in the experimental group.

Reference test: All participants with angiocardiography-proven CAS will be considered as controls.

#### 2.2.4 Type of outcome measurements

The primary outcomes are sensitivity and specificity. The secondary outcomes are positive likelihood ratio, negative likelihood ratio, and diagnostic odds ratio.

### 2.3 Data sources and search strategy

#### 2.3.1 Electronic searches

The literature search will be performed in electronic databases of MEDLINE, EMBASE, Cochrane Library, PSYCINFO, Web of Science, Allied and Complementary Medicine Database, and China National Knowledge Infrastructure. All electronic databases search will cover from the initial of indexing to the present with no restrictions of language and publication status. We will create an example of search strategy for MEDLINE in Table 1. In addition, we will also adapt similar search strategies to the other electronic databases.

**Table 1:**
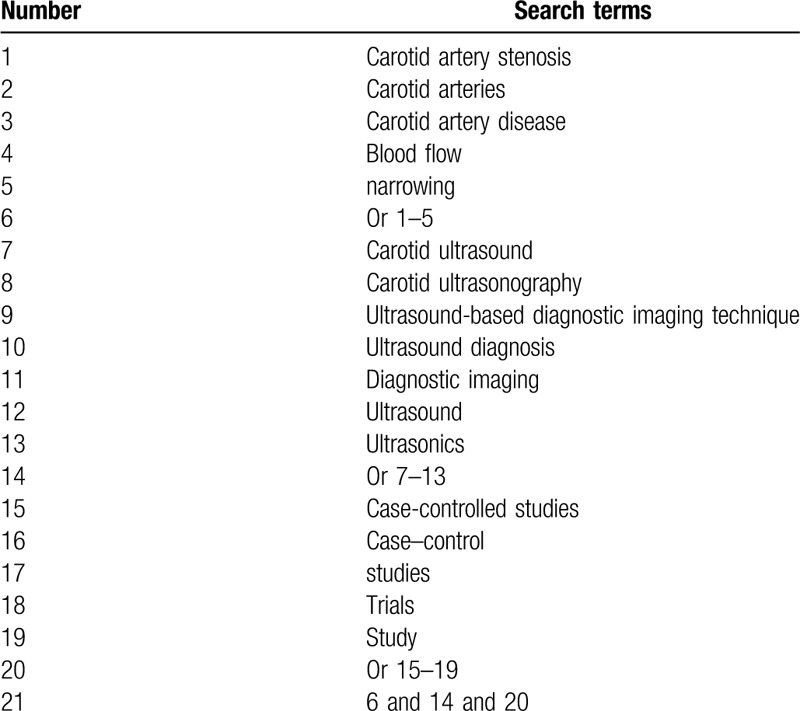
Search strategy for MEDLINE database.

#### 2.3.2 Other resources

In addition, we will also check conference abstracts, ongoing clinical trials from clinical trial registry, and reference lists of associated reviews.

### 2.4 Study selection and data collection

#### 2.4.1 Study selection

After identification of records from searching electronic databases, 2 authors will independently screen all titles and abstracts based on the predefined eligibility criteria. All irreverent records will be excluded. Then, the full text papers of all potential studies will be obtained, and we will apply full inclusion criteria to check if they meet all of them. In event of any confusion, a third author will help to resolve them by discussion. A detailed flow chart will be utilized to show the study selection process.

#### 2.4.2 Data extraction

Two authors will independently extract data using a standard data collection form designed before this study. Any conflicts between 2 authors will be solved by a third author through discussion. The extracted form includes information of first author, time of publication, country, race, age, sex, diagnostic criteria, eligibility criteria, sample size, study setting, study methods, index and reference tests, outcomes, and funding information. If there is insufficient or unclear data, we will contact primary authors to require it.

### 2.5 Quality assessment

The Quality Assessment of Diagnostic Accuracy Studies tool will be used for assessing study quality.^[23]^ Two independent authors will identify study quality for each included study. Any difficulties encountered between 2 authors will be discussed with a third author.

### 2.6 Assessment of heterogeneity

We will check heterogeneity among included studies using *I*^2^ statistic. A value of *I*^2^ ≤50% shows acceptable heterogeneity, and a Mantel–Haenszel fixed-effects model will be used. Otherwise, a value of *I*^2^ >50% exerts obvious heterogeneity, and a Mantel–Haenszel random-effects model will be utilized.

### 2.7 Statistical analysis

#### 2.7.1 Data synthesis

In this study, we will apply RevMan V.5.3 software: organization, Cochrane Community; city, London; country, UK and Stata V.12.0 software: organization, StataCorp; city, College Station; country, USA to analyze data. We will express outcome data as descriptive statistics and 95% confidence intervals. We will also plan to perform a descriptive forest plot and a summary receiver operating characteristic plot. If there is acceptable heterogeneity among included studies, we will plan to conduct a meta-analysis. Otherwise, we will perform a subgroup analysis to check the sources of obvious heterogeneity. If it is possible, we will also carry out narrative summary to synthesize outcome data.

#### 2.7.2 Subgroup analysis

We will perform a subgroup analysis to identify any possible sources for obvious heterogeneity according to the different types of study characteristics, indexes, and outcomes.

#### 2.7.3 Sensitivity analysis

We will also conduct a sensitivity analysis to check the robustness of outcome results by removing low quality studies.

#### 2.7.4 Reporting bias

If sufficient studies are included, we will check reporting bias using funnel plots and associated regression tests.^[24,25]^

### 2.8 Ethics and dissemination

This study will not need ethic approval as primary data will not be collected. The findings of this study will be disseminated though a relevant peer-reviewed journal or a conference.

## 3 Discussion

This study will be the first to explore the performance of existing evidence of early diagnosis of CAS using CU. This study will check the current evidence existence and assess their methodological strengths. It is expected that the findings of this study will be an essential step towards informing the selection of early diagnosis of CAS using CU both in clinical practice and health-related policy making.

## Author contributions

**Conceptualization:** Li-wei Qin, Li-hong Qin, Yun Yu, Xin-wei Hou, Chen Wang.

**Data curation:** Li-wei Qin, Li-hong Qin, Yun Yu, Xin-wei Hou, Chen Wang, Weeks Christina.

**Formal analysis:** Li-hong Qin, Chen Wang.

**Methodology:** Li-wei Qin, Li-hong Qin, Yun Yu, Xin-wei Hou, Chen Wang.

**Project administration:** Yun Yu.

**Resources:** Li-wei Qin, Li-hong Qin, Yun Yu, Xin-wei Hou, Chen Wang, Weeks Christina.

**Software:** Li-hong Qin, Xin-wei Hou, Chen Wang.

**Validation:** Li-wei Qin, Li-hong Qin, Yun Yu, Xin-wei Hou, Chen Wang, Weeks Christina.

**Visualization:** Li-wei Qin, Li-hong Qin, Yun Yu, Chen Wang, Weeks Christina.

**Writing – original draft:** Li-wei Qin, Li-hong Qin, Yun Yu, Xin-wei Hou, Chen Wang, Weeks Christina.

**Writing – review & editing:** Li-wei Qin, Li-hong Qin, Xin-wei Hou, Chen Wang, Weeks Christina.
